# Association between body mass index and patient-reported-outcome questionnaire scores (CAT™, ACT™, mMRC dyspnoea scale, IPAQ) in Ukraine, Kazakhstan and Azerbaijan: results of the CORE study

**DOI:** 10.1186/s12890-021-01542-2

**Published:** 2021-06-07

**Authors:** D. Nugmanova, Y. Feshchenko, L. Iashyna, M. Polianska, K. Malynovska, I. Stafeyeva, J. Makarova, A. Vasylyev

**Affiliations:** 1 Semey State Medical University, Almaty, Kazakhstan; 2 National Institute of Phtysiology and Pulmonology Named After F.G. Yanovskiy NA, Kyiv, Ukraine; 3 GlaxoSmithKline, Kiev, Ukraine; 4 GlaxoSmithKline, Moscow, 125167 Russia; 5 GlaxoSmithKline, Dubai, UAE

**Keywords:** Body mass index, COPD assessment test™, Modified medical research council dyspnoea, Asthma control test™, International physical activity questionnaire, Ukraine, Kazakhstan, Azerbaijan

## Abstract

**Background:**

The overweight/obese population (evaluated by a body mass index, BMI) represents a global health problem and contributes to the development of various chronic diseases. In this epidemiological study we evaluated this relationship by analyzing patient-reported questionnaires related to respiratory function, physical activity and BMI.

**Methods:**

In 2013–2015, adult residents of selected cities were enrolled to this study in: Ukraine (M/F: 403/561), Kazakhstan (M/F = 348/597) and Azerbaijan (M/F: 389/544). Height was measured using a vertical measuring board, and body weight was measured by using portable digital scales. All participants were interviewed using CAT™, mMRC scale and IPAQ; respondents who also reported wheezing or whistling chest sounds during the previous 12 months additionally ACT™.

**Results:**

45.4% of respondents in Ukraine, 47.6% in Kazakhstan and 54.9% of respondents in Azerbaijan were found to be overweight/obese (BMI ≥ 25 kg/m^2^). The mean CAT™ total score among this population versus those respondents with a normal weight was 5.2 versus 3.6 (Ukraine, *p* < 0.001), 4.2 versus 2.9 (Kazakhstan, *p* < 0.001) and 5.9 versus 4.3 (Azerbaijan, *p* < 0.001). The number of respondents without airflow limitations (mMRC score 0) among overweight/obese respondents versus normal weight respondents was 298 (68.2%) versus 456 (86.7%) in Ukraine, 261 (58.1%) versus 387 (78.2%) in Kazakhstan and 343 (67.1%) versus 345 (82.3%) in Azerbaijan. The ACT™ total score between overweight/obese respondents and normal weight respondents was not statistically different. IPAQ showed a tendency towards a higher proportion of “low activity” results (compared to “moderate” and “high”) in the overweight/obese subgroup (24.7% vs. 23.8% in Kazakhstan, 18.5% vs. 14.6% in Azerbaijan), and in Ukraine this difference was significant (12.4% vs. 5.2%, *p* < 0.001).

**Conclusion:**

CAT™ and mMRC are widely used tools for respiratory function assessment. Despite CAT™ scores being close to a normal value (< 5), the relationship of both CAT™ and mMRC scores with being overweight/obese was demonstrated in the general adult population of three CIS countries. IPAQ may also be a useful instrument for measuring activity level however, more objective studies are required to evaluate the relationship between BMI and physical activity.

**Supplementary Information:**

The online version contains supplementary material available at 10.1186/s12890-021-01542-2.

## Background

Obesity is a global health problem around the world and its prevalence has been increasing over several decades. Body mass index (BMI) is a simple index of weight-for-height that is commonly used to classify overweight and obesity in adults. The World Health Organization defines being overweight as a BMI ≥ 25 kg/m^2^ and obesity as a BMI ≥ 30 kg/m^2^) [1]. In 2016, more than 1.9 billion adults aged 18 years and older were overweight, which amounts to 39% of the adult population; of these over 650 million were obese, amounting to 13% of the adult population [1]. In 2010, high BMI was ranked sixth in the risk factors for global burden of disease, accounting for almost 3.5 million deaths per year [2].

It is well established that being overweight or obese can contribute to the development of various chronic diseases including; cardiovascular disease, metabolic syndrome, diabetes, osteoarthritis, and several types of malignancies [1, 3]. At the same time, relationship between BMI and chronic respiratory diseases is complex and an object of discussion. Obesity has been found to be strongly linked with respiratory symptoms and diseases including; exertional dyspnea, obstructive sleep apnea syndrome, obesity, hypoventilation syndrome, chronic obstructive pulmonary disease (COPD), asthma, pulmonary embolism, and aspiration pneumonia [4, 5]. The abdominal type of obesity can also play a crucial role in the development of lung function impairment and metabolic syndrome [6]. Some authors [7] suggest that being overweight or obese could also be a factor leading to misdiagnosis and subsequent overtreatment of COPD.

Furthermore, overweight and obese individuals are more likely to have respiratory symptoms than individuals with a normal BMI, even in the absence of demonstrable lung disease [8]. Being overweight or obese is also associated with a dose-dependent increase in the odds of asthma incidence in both men and women [9].

On the other hand, there is an inverse relationship between BMI and forced expiratory volume in one second (FEV_1_). BMI is also an independent prognostic factor for COPD, with a clear association between a low BMI and increased mortality. Several studies showed that the BMI/airflow obstruction/dyspnea/exercise capacity (BODE) index, a simple multidimensional grading system, is better than FEV_1_ at predicting the risk of death from any cause and from respiratory causes among patients with COPD [10, 11] Among patients hospitalized due to acute exacerbation of COPD, where low BMI is quite frequent, higher BMI was independently predictive of better long-term survival [12]. The reasons for this inverse effect include the mechanical effects of truncal obesity and the metabolic effects of adipose tissue [3].

This study was aimed to evaluate the results of different patient-reported questionnaires related to COPD and asthma (the COPD Assessment Test (CAT™) [13], the modified Medical Research Council (mMRC) dyspnoea scale [14], the Asthma Control Test (ACT™) [15] and the International Physical Activity Questionnaire (IPAQ) [16]) among the adult population of three Commonwealth of Independent States (CIS) countries, dependent on BMI. This evaluation was part of the cross-sectional CORE study, where the main goal was to assess the point prevalence of COPD, asthma and allergic rhinitis (AR) in these countries [17].

## Methods

### Study area and population

This cross-sectional epidemiological study was carried out in 2013–2015 across major cities of three countries: Ukraine (Kyiv), Kazakhstan (Almaty) and Azerbaijan (Baku). Data were captured during household visits, as described earlier [17].

The study enrolled 964 (90.4% of potentially eligible) participants in Ukraine, 945 (85.4% of potentially eligible) participants in Kazakhstan and 933 (96.9% of potentially eligible) participants in Azerbaijan. Inclusion criteria were: adults (≥ 18 years old), ≥ 10 years of residence in the city and written informed consent. People with contraindications to spirometry or bronchodilator administration, or who did not answer the study questionnaire were excluded.

### Patient-reported-outcomes questionnaires and BMI measurement

Socio-demographic data were captured from each participant. Height was measured with a vertical measuring board and recorded to the nearest 0.1 cm. Body weight was measured by using portable, strain gauge digital scales and recorded to the nearest 0.1 kg. BMI was calculated during statistical analysis as body weight (kg)/[height (m)]^2^.

During the visit the investigators performed spirometry testing for participants (spirometer: EasyOne™, NDD Medical Technologies, USA, provided by GlaxoSmithKline), without bronchodilator use (pre-dose) and 15–20 min later (post-dose, after inhalation of salbutamol 200–400 mcg (GlaxoSmithKline)). Participants were asked to fill in the ATS Respiratory Symptoms Questionnaire [18], CAT™ [13], ACT™ [15], the Alcohol Intake and Tobacco Smoking Questions and the IPAQ questionnaire [16]. Dyspnoea was evaluated using the mMRC dyspnoea scale [14]. These questionnaires are presented in Additional file 1.

### Criteria for COPD diagnosis

Criteria for COPD were predefined by the protocol using a standard spirometric criteria [19], when FEV_1_ (forced expiratory volume in one second): FVC (forced vital capacity) ratio ≥ 0.70 meant the absence of COPD. Study Executive Committee reviewed the quality and results of spirometry. Diagnosis of asthma was based on the Global Initiative for Asthma [20] Guidelines, using the ATS Respiratory Symptoms Questionnaire [18]. AR was diagnosed based on the self-reported (as highlighted in the ATS Respiratory Symptoms Questionnaire) presence of watery runny nose symptoms during the last 12 months, either alone or in combination with any of the following: nasal or ocular symptoms, sneezing, nasal obstruction, nasal itching, or conjunctivitis [21, 22].

The point prevalence of COPD, asthma and AR, including previously diagnosed and firstly diagnosed cases, was described earlier [17].

### Statistical analysis

For each country, data were analysed using IBM SPSS Statistics software (IBM Corp., USA) version 21.0 and R software version 3.1.2 (R Core Team, Austria). The number of overweight/obese individuals was divided by the total sample and multiplied by 1000, to estimate the point prevalence, together with 95% Clopper–Pearson confidence intervals (CI) [23]. Comparisons between categorical variables (IPAQ short form activity category) were performed using Chi-square criteria, between numerical variables using the Mann–Whitney test; statistical significant differences were established at *p* < 0.05. Missing data points were not imputed.

### Sample size justification

The number of subjects was based on precision approach for evaluation of prevalence. Taking into account a design effect of 1.25, expected prevalence of 10% with a half-width of 95% CI ± 3% or prevalence of 15–20% with a half-width of 95% CI ± 4%, sample size of 465 subjects was required. Subsequently, 930 evaluable individuals (465 aged 18–39 years old and 465 aged ≥ 40 years old) in each country had to be included for final analysis.

## Results

### Patient characteristics

Across three countries the proportion of participating males was slightly lower than females which reflected the census data proportions: 561 (58.2%) females and 403 (41.8%) males in Ukraine; 597 (63.2%) females and 348 (36.8%) males in Kazakhstan; 544 (58.3%) females and 389 (41.7%) males in Azerbaijan. All respondents in Azerbaijan and 99.7% in Ukraine were Caucasians, whereas in Kazakhstan 62.8% were Asians. Mean age of participants was 40.7 years (SD 15.1; 18.0–85.0) in Ukraine, 42.5 years (SD 15.3; 18.0–89.0) in Kazakhstan and 40.7 years (SD 14.8; 18.0–90.0) in Azerbaijan (Table 1).

**Table 1:** Demographic characteristics of sample respondents

	Ukraine	Kazakhstan	Azerbaijan
Age, years			
Mean (SD)	40.7 (15.1)	42.5 (15.3)	40.7 (14.8)
Min–Max	18.0–85.0	18.0–89.0	18.0–90.0
Sex, n (%)			
Male	403 (41.8%)	348 (36.8%)	389 (41.7%)
Female	561 (58.2%)	597 (63.2%)	544 (58.3%)
Total	964 (100.0%)	945 (100.0%)	933 (100.0%)
Ethnicity, n (%)			
Asian	3 (0.3%)	593 (62.8%)	0
Black	0	1 (0.1%)	0
Caucasian/ White	961 (99.7%)	349 (36.9%)	933 (100.0%)
Other	0	2 (0.2%)	0
Total	964 (100.0%)	945 (100.0%)	933 (100.0%)
BMI, kg/m^2^ (overall population)			
Mean (SD)	25.0 (5.1)	25.7 (5.1)	26.4 (5.3)
Min–Max	15.8–51.7	15.8–43.4	13.9–48.8
BMI category, n (%)			
< 25 kg/m^2^	526 (54.6%)	495 (52.4%)	419 (45.1%)
≥ 25 kg/m^2^	437 (45.4%)	449 (47.6%)	511 (54.9%)
Total	963 (100.0%)	944 (100.0%)	930 (100.0%)
BMI, kg/m^2^, in males			
Mean (SD)	25.5 (4.3)	25.7 (4.4)	26.0 (4.5)
BMI ≥ 25, n/N (%)	210/403 (52.1%)	165/347 (47.6%)	212/389 (54.5%)
BMI, kg/m^2^, in females			
Mean (SD)	24.7 (5.5)	25.6 (5.5)	26.7 (5.8)
BMI ≥ 25, n/N (%)	227/560 (40.5%)	284/597 (47.6%)	299/541 (55.3%)
Smoking status, n/N (%)			
Never smoked	629/954 (65.9%)	564/944 (59.7%)	690/933 (74.0%)
Current/past smoker	325/954 (34.1%)	380/944 (40.3%)	243/933 (26.0%)
COPD diagnosis (post-bronchodilator FEV_1_/FVC < 0.7), n/N (%)	30/939 (3.2%)	63/945 (6.7%)	35/933 (3.8%)
FEV_1_/FVC < 0.7 in males, n/N (%)	18/395 (4.6%)	42/348 (12.1%)	21/389 (5.4%)
FEV_1_/FVC < 0.7 in females, n/N (%)	12/544 (2.2%)	21/597 (3.5%)	14/544 (2.6%)
Stage I: Mild, n/N (%)	13/939 (1.4%)	26/945 (2.8%)	13/933 (1.4%)
Stage II: Moderate, n/N (%)	17/939 (1.8%)	37/945 (3.9%)	22/933 (2.4%)
Bronchial asthma (based on reported wheezing), n/N (%)	70/941 (7.4%)	237/930 (25.5%)	115/932 (12.3%)
Allergic rhinitis (by definition*), n/N (%)	43/963 (4.5%)	92/945 (9.7%)	80/933 (8.6%)

* running nose + 1 nasal or ocular symptom

**Fig. 1:**
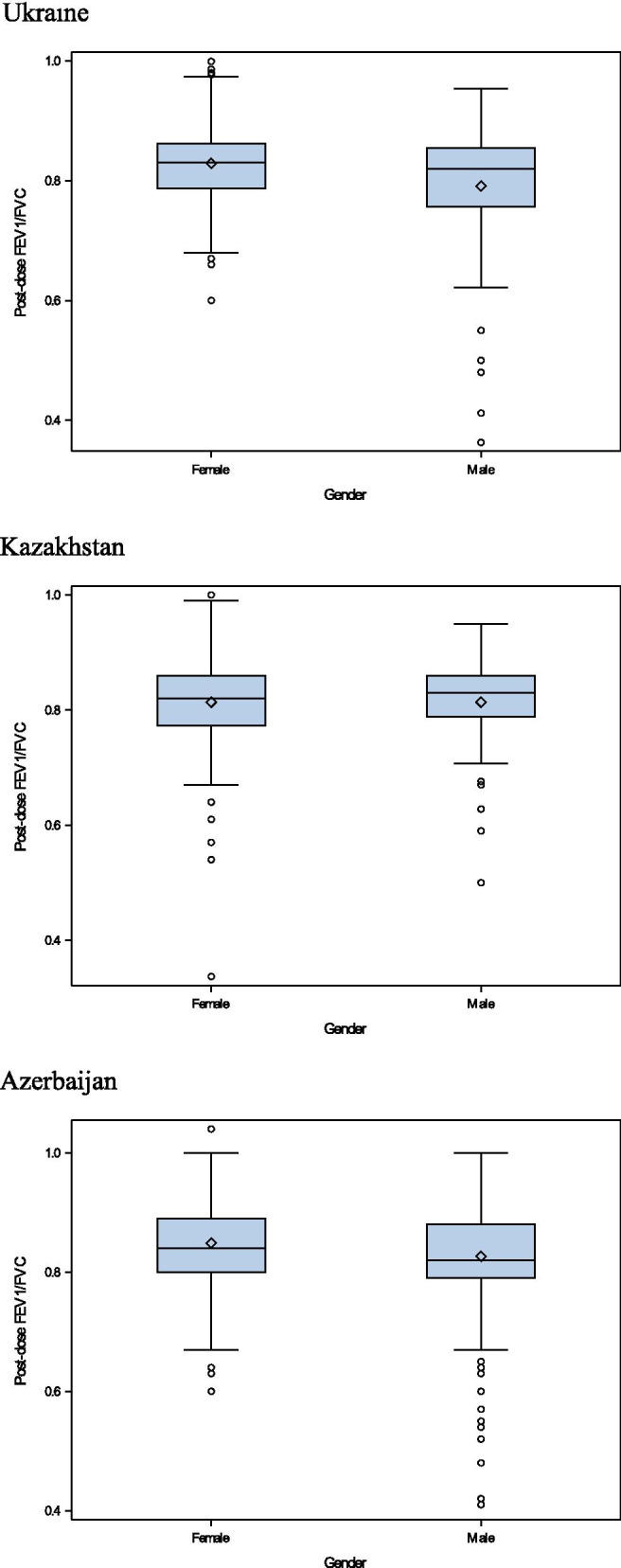
Bronchodilator response (post-dose FEV_1_/FVC) by sex (left: females, right: males)

About half of the respondents were overweight (or obese), i.e. BMI > 25 kg/m^2^, with the following distribution between countries: 437 (45.4%) in Ukraine; 449 (47.6%) in Kazakhstan and 511 (54.9%) in Azerbaijan. COPD based on spirometry results was diagnosed in 3.2% of respondents in Ukraine; 3.8% of respondents in Azerbaijan and almost twice as frequently in Kazakhstan at 6.7% of respondents. Moderate stage of COPD (by GOLD classification) was predominant. In male population, the percent of respondents with COPD (post-dose FEV_1_/FVC < 0.7) was higher than in females (Table 1). The graphs also show that mean FEV_1_/FVC values in males are lower than in females in Kazakhstan and Azerbaijan (Fig. 1). Asthma was diagnosed in 7.4% of respondents in Ukraine, 12.3% in Azerbaijan and 25.5% in Kazakhstan. AR was diagnosed in 4.5%, 8.6% and 9.7% of respondents, correspondingly (Table 1).

### Point prevalence of overweight/obesity

Point prevalence of being overweight/obese was similar in Ukraine (453.8 per 1000 persons (95% CI 422.0–485.9)) and Kazakhstan (475.6 per 1000 persons (95% CI 443.4–508.1)) and seemed to be higher in Azerbaijan (549.5 per 1000 persons (95% CI 516.8–581.8)). See Table 2.

**Table 2:** Point-prevalence of overweight/obesity (BMI > 25 kg/m^2^) in adult population

	Ukraine				Kazakhstan				Azerbaijan			
	Outcomes	Population	Prevalence per 1000	95% CI	Outcomes	Population	Prevalence per 1000	95% CI	Outcomes	Population	Prevalence per 1000	95% CI
Overall population	437	963	453.8	422.0–485.9	449	944	475.6	443.4–508.1	511	930	549.5	516.8–581.8
Males	210	403	521.1	471.1–570.8	165	347	475.5	421.9–529.5	212	389	545.0	494.0–595.3
Females	227	560	405.4	364.4–447.3	284	597	475.7	435.0–516.7	299	541	552.7	509.7–595.1

### Patient-reported questionnaires

Overweight/obese respondents had higher CAT™ total score compared to normal weight persons. In Ukraine, the mean (± SD) CAT™ total score was 5.2 ± 6.0 (median of 3.0) in overweight/obese respondents versus 3.6 ± 5.0 (median of 2.0) in respondents with normal weight (*p* < 0.001). In Kazakhstan the mean CAT™ total score was 4.2 ± 4.4 (median of 3.0) versus 2.9 ± 4.2 (median of 1.0) (*p* < 0.001) and in Azerbaijan it was 5.9 ± 5.9 (median of 4.0) and 4.3 ± 5.1 (median of 3.0) in respondents who were overweight/obese and normal weight, respectively (*p* < 0.001). See Table 3.

**Table 3:** COPD Assessment Test™ score in respondents with normal weight (BMI < 25 kg/m^2^) and overweight/obesity (BMI ≥ 25 kg/m^2^)

	Ukraine			Kazakhstan			Azerbaijan		
CAT™ total score	Overall population	< 25 kg/m^2^	≥ 25 kg/m^2^	Overall population	< 25 kg/m^2^	≥ 25 kg/m^2^	Overall population	< 25 kg/m^2^	≥ 25 kg/m^2^
N	963	526	436	944	494	449	933	419	511
Mean	4.3	3.6	5.2	3.5	2.9	4.2	5.2	4.3	5.9
SD	5.5	5.0	6.0	4.4	4.2	4.4	5.7	5.1	5.9
Median	2.0	2.0	3.0	2.0	1.0	3.0	3.0	3.0	4.0
Min–Max	0.0–32.0	0.0–32.0	0.0–31.0	0.0–27.0	0.0–27.0	0.0–23.0	0.0–39.0	0.0–39.0	0.0–29.0
*p* value	–	< 0.001		–	< 0.001		–	< 0.001	

Similarly to the CAT™, the mMRC dyspnoea scores were different depending on BMI category. In Ukraine, the percent of subjects without airflow limitations (mMRC score 0) was lower among respondents who were overweight/obese compared with participants who were normal weight. The number of respondents with an mMRC score of 0 was 298 (68.2%) among respondents who were overweight/obese and 456 (86.7%) among respondents with a normal weight. In Kazakhstan, 261 (58.1%) overweight/obese and 387 (78.2%) normal weight respondents had mMRC score of 0, and in Azerbaijan 343 (67.1%) overweight/obese and 345 (82.3%) normal weight respondents had mMRC score of 0. Correspondingly, the number of respondents with mild and moderate dyspnoea (mMRC scores 1 and 2) was higher among respondents who were overweight/obese compared with participants with a normal weight. There were significant differences in mMRC score between respondents who were overweight/obese and normal weight across all three countries (*p* < 0.001). See Table 4.

**Table 4:** Modified Medical Research Council (mMRC) dyspnoea score in respondents with normal weight (BMI < 25 kg/m^2^) and overweight/obesity (BMI ≥ 25 kg/m^2^)

mMRC dyspnoea score*	Ukraine			Kazakhstan			Azerbaijan		
	Overall population	< 25 kg/m^2^	≥ 25 kg/m^2^	Overall population	< 25 kg/m^2^	≥ 25 kg/m^2^	Overall population	< 25 kg/m^2^	≥ 25 kg/m^2^
0	755 (78.3%)	456 (86.7%)	298 (68.2%)	648 (68.6%)	387 (78.2%)	261 (58.1%)	691 (74.1%)	345 (82.3%)	343 (67.1%)
1	164 (17.0%)	51 (9.7%)	113 (25.9%)	248 (26.2%)	87 (17.6%)	160 (35.6%)	211 (22.6%)	66 (15.8%)	145 (28.4%)
2	24 (2.5%)	10 (1.9%)	14 (3.2%)	37 (3.9%)	13 (2.6%)	24 (5.3%)	19 (2.0%)	4 (1.0%)	15 (2.9%)
3	9 (0.9%)	4 (0.8%)	5 (1.1%)	9 (1.0%)	5 (1.0%)	4 (0.9%)	7 (0.8%)	2 (0.5%)	5 (1.0%)
4	5 (0.5%)	2 (0.4%)	3 (0.7%)	0 (0.0%)	0 (0.0%)	0 (0.0%)	0 (0.0%)	0 (0.0%)	0 (0.0%)
Missing	7 (0.7%)	3 (0.6%)	4 (0.9%)	3 (0.3%)	3 (0.6%)	0 (0.0%)	5 (0.5%)	2 (0.5%)	3 (0.6%)
Total	964 (100.0%)	526 (100.0%)	437 (100.0%)	945 (100.0%)	495 (100.0%)	449 (100.0%)	933 (100,0%)	419 (100.0%)	511 (100.0%)
*p* value	–	< 0.001		–	< 0.001		–	< 0.001	

*mMRC dyspnoea scores:

0: Responder is not affected by shortness of breath, except when engaging in strenuous exercise

1: Responder has shortness of breath when walking briskly on flat ground or slightly uphill

2: Responder walks more slowly on flat surfaces than other people his/her age because of shortness of breath, or he/she has to stop to catch the breath when walking at his/her own pace on flat ground

3: Responder has to stop to catch his/her breath after walking around 100 m or after walking for a few minutes on flat ground

4: Responder’s shortness of breath prevents him/her from leaving home or he/she has shortness of breath when dressing or undressing

ACT™ was filled in by any respondents who experienced wheezing or whistling sounds in their chest during the last 12 months. There were no significant differences in the ACT™ total score between respondents who were overweight/obese and normal weight in Ukraine (*p* = 0.800), Kazakhstan (*p* = 0.207) or Azerbaijan (*p* = 0.836). However, as the population size for this group was small, results should be interpreted with caution. See Table 5.

**Table 5:** Asthma Control Test™ score in respondents with normal weight (BMI < 25 kg/m^2^) and overweight/obesity (BMI ≥ 25 kg/m^2^)

ACT™ total score	Ukraine			Kazakhstan			Azerbaijan		
	Overall population	< 25 kg/m^2^	≥ 25 kg/m^2^	Overall population	< 25 kg/m^2^	≥ 25 kg/m^2^	Overall population	< 25 kg/m^2^	≥ 25 kg/m^2^
N	5	2	3	95	36	59	13	6	7
Mean (SD)	19.8 (5.5)	20.5 (3.5)	19.3 (7.2)	23.4 (3.1)	22.9 (3.5)	23.8 (2.8)	17.0 (4.7)	17.3 (4.6)	16.7 (5.1)
Median	23.0	20.5	23.0	25.0	25.0	25.0	16.0	19.5	16.0
*p* value	–	0.800		–	0.207		–	0.836	

The IPAQ questionnaire showed that in Ukraine the distribution of categories of physical activity (low/moderate/high) was significantly different between respondents with a normal weight and those who were overweight/obese. Thus, the proportion of respondents with a low activity level was higher among persons who were overweight/obese (12.4%) compared to those with normal weight (5.2%; *p* < 0.001). There were no significant differences in the distribution of categories of physical activity in Kazakhstan (*p* = 0.481) as well as in Azerbaijan (*p* = 0.117). Similarly, the MET-min per week parameter for walking was significantly higher among respondents with a normal weight compared to those who were overweight/obese in Ukraine and Azerbaijan. In Ukraine, MET-min per week for walking (mean ± SD) was 2144.3 ± 1609.1 in respondents with normal weight and 1906.6 ± 1501.9 in respondents who were overweight/obese (*p* = 0.031). In Azerbaijan, MET-min per week for walking was 2534.9 ± 1732.9 in respondents with normal weight and 2136.7 ± 1716.1 in respondents wo were overweight/obese (*p* < 0.001). In Kazakhstan, MET-min per week parameters did not significantly differ between respondents who were normal weight and either overweight/obese. See Table 6.

**Table 6:** Results of International Physical Activity Questionnaire (IPAQ) in respondents with normal weight (BMI < 25 kg/m^2^) and overweight/obesity (BMI ≥ 25 kg/m^2^)

IPAQ short form activity	Ukraine			Kazakhstan			Azerbaijan		
	Overall population	< 25 kg/m^2^	≥ 25 kg/m^2^	Overall population	< 25 kg/m^2^	≥ 25 kg/m^2^	Overall population	< 25 kg/m^2^	≥ 25 kg/m^2^
Categories									
Inactive (low)	80 (8.4%)	27 (5.2%)	53 (12.4%)	230 (24.3%)	118 (23.8%)	111 (24.7%)	155 (16.7%)	61 (14.6%)	94 (18.5%)
Moderate	357 (37.7%)	194 (37.3%)	162 (37.9%)	377 (39.9%)	191 (38.6%)	186 (41.4%)	406 (43.7%)	178 (42.6%)	227 (44.6%)
High	511 (53.9%)	299 (57.5%)	212 (49.6%)	338 (35.8%)	186 (37.6%)	152 (33.9%)	369 (39.7%)	179 (42.8%)	188 (36.9%)
Total	948 (100.0%)	520 (100.0%)	427 (100.0%)	945 (100.0%)	495 (100.0%)	449 (100.0%)	930 (100.0%)	418 (100.0%)	509 (100.0%)
* p* value	–	< 0.001		–	0.481		–	0.117	
Walk MET-min per week									
N	923	510	412	847	445	402	873	396	474
Mean	2036.6	2144.3	1906.6	1729.7	1704.5	1757.6	2323.2	2534.9	2136.7
SD	1565.5	1609.1	1501.9	1516.7	1495.3	1541.5	1735.0	1732.9	1716.1
Median	1386.0	1386.0	1386.0	1386.0	1386.0	1386.0	1386.0	2772.0	1386.0
* p* value	–	0.031		–	0.644		–	< 0.001	
Mod MET-min per week									
N	749	427	321	440	240	200	284	133	151
Mean	2549.8	2602.6	2484.4	1805.5	1648.8	1993.6	2607.8	2520.8	2684.4
SD	2371.2	2412.2	2319.6	1843.2	1727.6	1960.9	2135.4	2224.0	2058.6
Median	1440.0	1440.0	1440.0	960.0	960.0	1440.0	1680.0	1680.0	1680.0
* p* value	–	0.578		–	0.059		–	0.259	
Vig MET-min per week									
N	581	349	231	189	106	83	79	41	38
Mean	1367.4	1415.1	1297.1	4053.5	3864.9	4294.5	7578.7	7262.4	7920.0
SD	2389.8	2446.5	2310.2	3818.6	3490.2	4210.6	4300.1	4297.9	4333.7
Median	480.0	480.0	240.0	2880.0	2880.0	2400.0	7680.0	6720.0	9600.0
* p* value	-	0.191		–	0.939		–	0.525	
Total MET-min/week									
N	928	515	412	883	464	419	892	398	491
Mean	4939.7	5240.4	4569.5	3426.5	3370.4	3488.6	3775.2	4112.6	3501.2
SD	4149.7	4236.5	4016.2	3557.8	3411.4	3716.3	3830.4	3917.1	3751.2
Median	3478.5	3804.0	3066.0	2190.0	2209.0	2175.0	2772.0	2772.0	2376.0
* p* value	–	0.004		–	0.943		–	0.001	

## Discussion

In our study several important factors related to respiratory function were evaluated among general adults with a normal weight and who were overweight/obese. This also included the impact these factors had on COPD (by CAT™), dyspnoea (by mMRC scale) and physical activity level (by IPAQ).

**Fig. 2:**
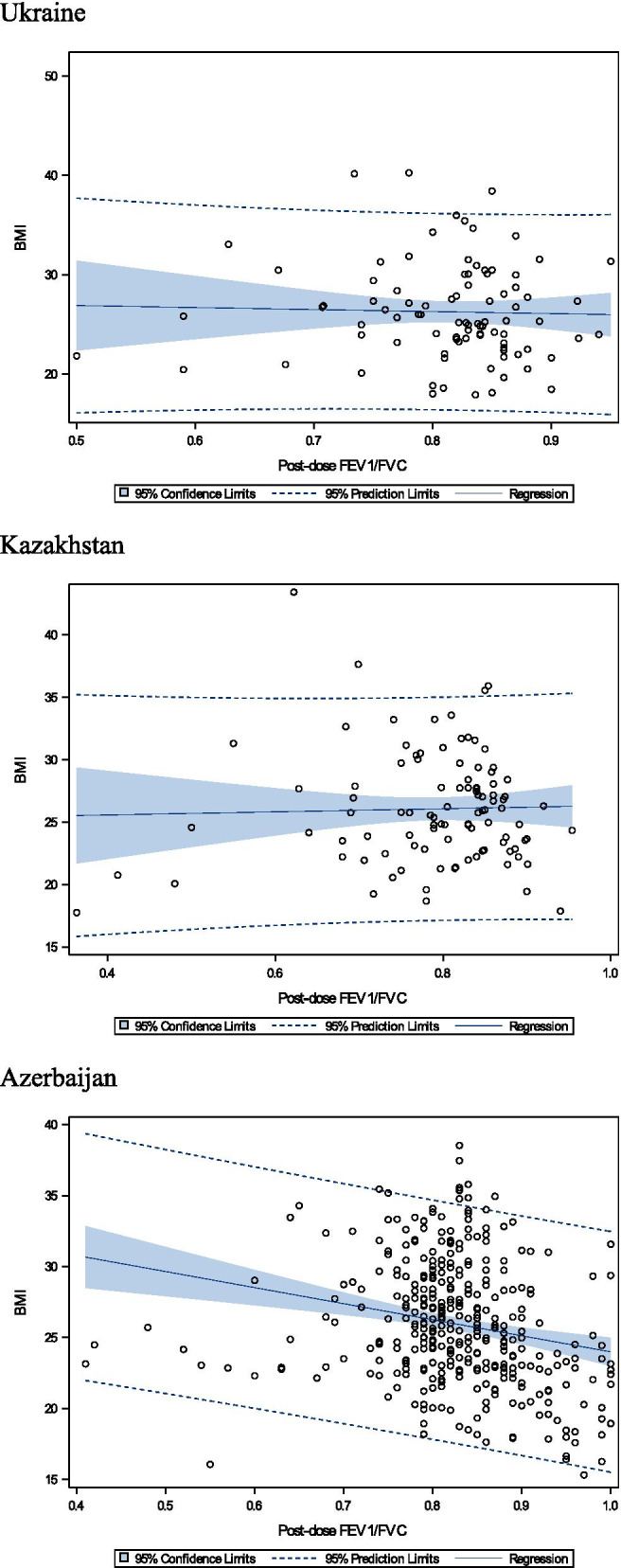
Relationship of post-dose FEV_1_/FVC values versus BMI, in males

**Fig. 3:**
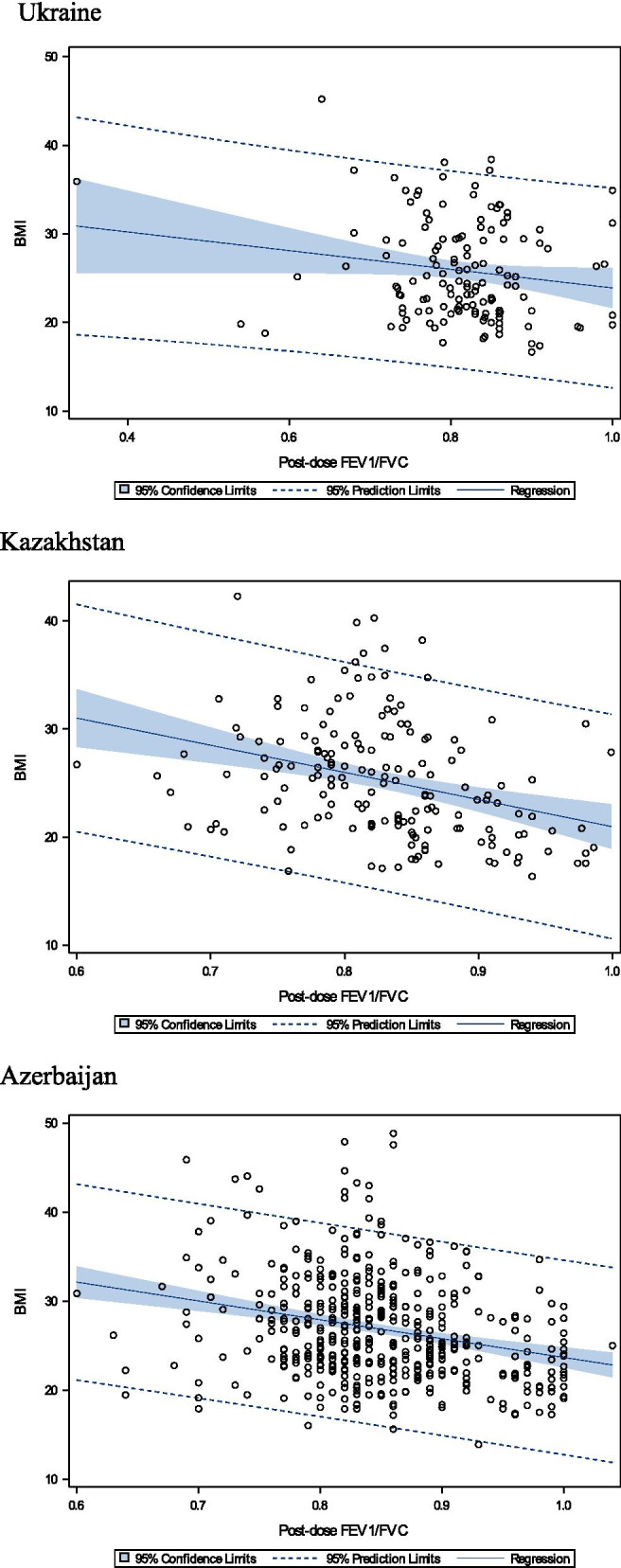
Relationship of post-dose FEV_1_/FVC values versus BMI, in females

The relationship of post-bronchodilator FEV_1_/FVC values and BMI differ by country and by sex, which may be explained by variability in occupational preferences of males and females, environmental, geographical, ethnicity and true biological phenomena, along with common risk factors, affecting lung function (smoking, dusty work etc.). The strongest relationship between the post-dose FEV_1_/FVC values and BMI was observed among Azerbaijan population (Figs. 2, 3).

In Kazakhstan the relationship of post-dose FEV_1_/FVC values versus BMI in both sexes was shifting to the left, indicating to potentially more harmful impact of associated risk factors on lung function. As shown on the Table 1 prevalence of COPD is substantially higher in Kazakhstan compared to other countries (6.7%). Prevalence of COPD in male population is more than 3 times higher to that among female in Kazakhstan. As was reported previously [24, 25] COPD diagnosis was associated with individual risk factors as smoking (OR 3.756 (CI 2.156–6.543) *p* < 0.001) and dusty work (OR 2.306 (CI 1.328–4.002) *p* = 0.002), which complements higher prevalence of smoking in Kazakhstan, compared to other countries (Table 1). Authors additionally assume other potential harmful factors, adversely impacting lung function, which include relatively poor ecological conditions in the city Almaty, Kazakhstan, caused by heavy air pollution aggravated by high mountains (3000–5000 m) surrounding the city, and little winds with no proximity to large bodies of water.

Despite the mean CAT™ score corresponding to a normal level in healthy non-smokers (< 5), a statistically significant difference was revealed with a higher CAT™ total score observed among respondents who were overweight/obese compared to those with a normal weight across three countries. CAT™ total score ranged between 0 and 27–39, i.e. maximal value corresponded to high and very high impact of COPD. Mean CAT™ total score in overweight/obese respondents in our study (from 4.2 to 5.9) was similar to that reported in the general health population as seen in the BREATHE study assessing the Arabic version (7.0) [26], the Japanese version of CAT™ (5.8) [27], and the CAT™ as assessed in Canada (6.0) [28]. Interestingly, in two cross-sectional studies assessing patients with stable COPD, the relationship between CAT™ score and BMI was not statistically significant [29, 30]. In Italy, also no significant correlation was found between the CAT™ score and sex, age, BMI, or the educational level of subjects with COPD with different severities [31]. As for the differences observed between overweight/obese persons and normal weight adults in the general population obtained from this study, it is doubtful if it could be considered as clinically significant as it was established earlier that the most reliable estimate of the minimum important difference of the CAT™ is 2 points [32]. However, the differences between the mean values of CAT™ total score in our study were 1.6 in both Ukraine and Azerbaijan and 1.3 in Kazakhstan.

Dyspnea is common in obese subjects. However, its assessment is complex in clinical practice. It is known that mMRC scale is one of the instruments that had been tested and can be recommended for use in people with increased adiposity based on its psychometric properties (reliability (correlations > 0.8)) and concurrent validity (correlation with severity of airways obstruction and walking distance). Other instruments to assess dyspnea include the Visual Analog Scale, Modified Borg Scale, and Baseline Dyspnea Index (BDI) [33, 34]. In the CORE study, the results of the mMRC scale in a general adult population confirmed the data obtained in other studies, showing a higher prevalence of dyspnea among persons who were overweight/obese compared to those with a normal weight; the number of "non dyspneic" participants was 68.2% among respondents who were overweight/obese and 86.7% among respondents with normal weight. In the study conducted by Launois et al. [35], among 45 obese subjects studied, 84% patients had an mMRC score of ≥ 1 and 40% had an mMRC ≥ 2; differences were obtained between the "dyspneic" and the "non dyspneic" groups as assessed by the mMRC scale for BMI, expiratory reserve volume (ERV), FEV_1_ and distance covered in 6-min walk test (6MWT) [35].

As for physical activity, in our study it was shown that BMI had influence over the distribution of categories of physical activity (low/moderate/high) in a general adult population. In Ukraine the rate of inactivity was 12.4% among overweight/obese persons and 5.2% among normal weight persons (*p* < 0.001). In Kazakhstan and Azerbaijan the proportion of persons with inactivity was higher among those who were overweight/obese compared to normal weight persons (24.7% vs. 23.8% in Kazakhstan, 18.5% vs. 14.6% in Azerbaijan), but the differences were not statistically significant.

IPAQ is a widely used instrument to measure physical activity level in the general health population. In a Spanish study among Colombian college students, an excessive weight was observed in 26.47% of the students where an association between physical inactivity (assessed by IPAQ) and excessive weight was observed [36]. In another study with participation of students from Peru, according to the IPAQ, 53.9% of the participants recorded high levels of physical activity, 35.4% recorded moderate levels, and 10.7%, recorded low levels [37]. In a large Mexican survey [38] among adults it was shown that obesity, the 60–69 year age group and high socioeconomic status were related to more frequent physical inactivity; the prevalence of physical inactivity in 2012 was 19.4% (95% CI 18.1, 20.7). In the study of Tehard et al. [39] among 757 obese subjects, about one third of men and women were classified as insufficiently active by IPAQ. Therefore, the results of our study on the prevalence of inactivity and relationship with high BMI were in line with data obtained in other studies. However, IPAQ assesses physical activity which was self-reported by the CORE study participants. Therefore, obese and overweight persons could have a different meaning of “vigorous” or “moderate” activity than lean and normal weight people. For example, they may walk slower or shorter distances and report these activities as high level in comparison with normal BMI respondents. In the future, studies could include more objective assessment tools (watches/bracelets or other devices, with real time activity monitoring), which would demonstrate better correlation between BMI and physical activity level.

### Limitations of the study

This study was well-designed and conducted in a large population, according to an identical protocol in each country. Cross-sectional design allowed capturing data during a single visit. However, the study does have some limitations.

Despite the households were chosen based on two-step cluster randomization, selection bias cannot be fully excluded. Study population was presented by residents of major cities, and this fact limits the extrapolation of the study data to rural population or within a country. Finally, there might be insufficiently valid or missing data, if they were collected from participants’ word.

## Conclusions

In conclusion, CAT™ and mMRC are widely used tools for respiratory function assessment. Despite CAT™ scores being close to a normal value (< 5), the relationship of both CAT™ and mMRC scores with being overweight/obese was demonstrated in the general adult population of three CIS countries. IPAQ may also be a useful instrument for measuring activity level however, more objective studies are required to evaluate the relationship between BMI and physical activity.

## Supplementary Information

**Additional file 1**. “Study questionnaires used in the CORE study” contains the description of patient-reported questionnaires used in the CORE study.

## Data Availability

The datasets used and/or analysed during the current study are available from the corresponding author on reasonable request.

## Abbreviations

AR: Allergic rhinitis
BMI: Body mass index
CAT: COPD assessment test
CI: Confidence interval
CIS: Commonwealth of independent states
COPD: Chronic obstructive pulmonary disease
FEV_1_: Forced expiratory volume in one second
FVC: Forced vital capacity
GSK: GlaxoSmithKline
SD: Standard deviation
