# Supplementary material for: Association between body mass index and patient-reported-outcome questionnaire scores (CAT™, ACT™, mMRC dyspnoea scale, IPAQ) in Ukraine, Kazakhstan and Azerbaijan: results of the CORE study
Source: BMC Pulm Med. 2021 Jun 7;21:192. doi: 10.1186/s12890-021-01542-2 (PMC8182935; doi:10.1186/s12890-021-01542-2)
Supplement: Supplementary file 1 — Additional file 1. “Study questionnaires used in the CORE study” contains the description of patient-reported questionnaires used in the CORE study. [file 12890_2021_1542_MOESM1_ESM.docx]

**Additional file 1**

**Study questionnaires used in the CORE study**

**COPD Assessment Test (CAT™)** is a short validated patient-completed questionnaire, assessing the impact of COPD on health status. [1] It has good measurement properties, is sensitive to differences in state and provides a valid, reliable and standardized measure of COPD health status with worldwide relevance. It comprises 8 simple questions that most patients should be able to understand and answer easily (cough, phlegm (mucus), tightness in the chest, breathless during walking up, limitation doing any activities at home, confidence in leaving home despite a lung condition, sleeping soundly, amount of energy). The CAT™ has a scoring range of 0-40 (COPD impact level: >30 very high, >20 high, 10-20 medium, <10 low, and 5 – upper limit of normal in healthy non-smokers). CAT™ is available at [www.CATestonline.org](http://www.CATestonline.org).

**The modified Medical Research Council (mMRC) dyspnoea scale** uses a simple grading system to assess a patient's level of dyspnea – shortness of breath. [2] The degree of dyspnoea was rated as 0 (Responder is not affected by shortness of breath, except when engaging in strenuous exercise), 1 (Responder has shortness of breath when walking briskly on flat ground or slightly uphill), 2 (Responder walks more slowly on flat surfaces than other people his/her age because of shortness of breath, or he/she has to stop to catch the breath when walking at his/her own pace on flat ground), 3 (Responder has to stop to catch his/her breath after walking around 100 m or after walking for a few minutes on flat ground) and 4 (Responder’s shortness of breath prevents him/her from leaving home or he/she has shortness of breath when dressing or undressing).

**Asthma Control Test (ACT^TM^)** is a short, simple, patient-based tool for identifying patients with poorly controlled asthma. [3] The ACT^TM^ was clinically validated by specialist assessment and spirometry and is reliable, and responsive to changes in asthma control over time in patients new to the care of asthma specialists. The questionnaire consists of 5 questions about asthma symptoms, with their severity ranging by the 5-point scale. A cutoff score of 19 or less identifies patients with poorly controlled asthma. ACT^TM^ is available at [www.asthmacontroltest.com](http://www.asthmacontroltest.com).

**International Physical Activity Questionnaire (IPAQ)** was developed as an instrument for cross-national monitoring of physical activity and inactivity. IPAQ has reasonable measurement properties for monitoring population levels of physical activity among 18- to 65-years old adults in diverse settings. Extensive reliability and validity testing was undertaken across 12 countries during 2000 after developing this questionnaire. [4] The questionnaire comprises a set of 4 questionnaires. Long (5 activity domains asked independently) and short (4 generic items) versions for use by either telephone or self-administered methods are available. The short-form is recommended for national monitoring and was used in this study. IPAQ assesses physical activity undertaken across a comprehensive set of domains including: a. leisure time physical activity; b. domestic and gardening (yard) activities; c. work-related physical activity; and d. transport-related physical activity. The IPAQ short form asks about three specific types of activity undertaken in the four domains introduced above. The specific types of activity that are assessed are walking, moderate-intensity activities and vigorous-intensity activities. The outcomes from IPAQ is expressed categorically as low, moderate or high physical activity level. [5] Those individuals who not meet criteria for categories “moderate” or “high” are considered low/inactive. Respondents were assigned with moderate category in presence of any one of the following: 3 or more days of vigorous activity of at least 20 minutes per day OR 5 or more days of moderate-intensity activity or walking of at least 30 minutes per day OR 5 or more days of any combination of walking, moderate-intensity or vigorous intensity activities achieving a minimum of at least 600 MET-min/week. High activity was assigned in case of any of the following 2 criteria: Vigorous-intensity activity on at least 3 days and accumulating at least 1500 MET-minutes/week OR 7 or more days of any combination of walking, moderate-intensity or vigorous intensity activities achieving a minimum of at least 3000 MET-minutes/week. Continuous score is expressed as MET-min per week: MET level x minutes of activity x events per week. [5] IPAQ is available at [http://www.ipaq.ki.se](http://www.ipaq.ki.se/).

**ATS Respiratory Symptoms Questionnaire** was developed in 1978 in order to improve the respiratory questionnaires, which had been used earlier; it is an investigator-reported, close-ended questionnaire that is used for COPD, BA and AR case detection in the study population. [6] This questionnaire is recommended for use in epidemiologic studies of all respondents 13 or more years of age. It is divided into 2 components: the initial component is recommended as a minimal set of questions to be asked in every survey; these are followed by a group of optional questions that are left up to the discretion of the individual investigator to consider for inclusion at the end of the appropriate section of the questionnaire. The following sets of questions are included: cough, phlegm, wheezing, chest colds and chest illnesses. The questionnaire is available at <https://www.thoracic.org/statements/resources/archive/rrdquacer.pdf>.

**Alcohol Intake** **Questions** were developed for the purposes of this study. The respondent was asked to tick one answer:

- Does not take at all
- Moderate (up to 14 drinks per week for women and up to 21 drinks per week for men)
- Heavy or high-risk drinking (more than 3 drinks on any day or more than 14 per week for women and more than 4 drinks on any day or more than 21 per week for men)
- Binge drinking (consumption within 2 hours of 4 or more drinks for women and 5 or more drinks for men) a 'drink' refers to 15 ml of alcohol (e.g., one 360 ml. beer, one 150 ml glass of wine, or one 45 ml. Shot of distilled spirits)

**Tobacco Smoking Questions** consisted of 23 simple questions about smoking cigarettes in the present time and in the past:

- Have you ever smoked cigarettes? (No means less than 20 packs of cigarettes or 340 gr of tobacco in a lifetime or less than 1 cigarette a day for 1 year)
- Do you now smoke cigarettes (as of 1 month ago)?
- How old were you when you first started regular cigarette smoking?
- If you have stopped smoking cigarettes completely, how old were you when you stopped?
- How many cigarettes do you smoke per day now?
- On the average of the entire time you smoked, how many cigarettes did you smoke per day?
- Going back to your childhood if you ever lived with any person who used to smoke in your presence?
- How many cigarettes this person smoked per day?
- Did he/she smoke in your presence every day?
- How long did you live with this person?
- Does your spouse smoke?
- How many cigarettes this person smoked per day?
- Did he/she smoked in your presence every day?
- How long do you live with this person?
- Does any other member or your family smoke(d)?
- How many cigarettes this person smoke(d) per day?
- Did he/she smoke(d) in your presence every day?
- How long did you live with this person?
- Have you ever worked in an indoor place where you were exposed to tobacco smoke?
- How long did you worked at this place (if there were several places, please report total number of years)?
- Was this place very smoky?
- Have you ever (lifetime) travelled daily or at least a couple of times per week by car, train, bus or another vehicle which was smoky
- If you ever spent regularly (at least once a week) some time in a smoky place indoors other than at home or at work?

**References**:

1. Jones P, Harding G, Berry P, et al. Development and first validation of the COPD Assessment Test. European Respiratory Journal. 2009; 34(3): p. 648-654.
2. Schatz M, Sorkness C, Li J, Marcus P, Murray J, Nathan R, et al. Asthma Control Test: reliability, validity, and responsiveness in patients not previously followed by asthma specialists. J Allergy Clin Immunol. 2006; 117(3): p. 549-556.
3. Ekici A, Bulcun E, Karakoc T, Senturk E, Ekici M. Factors Associated With Quality of Life in Subjects With Stable COPD. Respir Care. 2015 Jul 7.
4. Craig C, Marshall A, Sjöström M, Bauman A, Booth M, Ainsworth B, et al. International physical activity questionnaire: 12-country reliability and validity. Med Sci Sports Exerc. 2003; 35(8): p. 1381-1395.
5. IPAQ scoring protocol - International Physical Activity Questionnaire [Internet]. [cited 2016 March]. Available from: <https://sites.google.com/site/theipaq/scoring-protocol>
6. Bellia V, Pistelli F, Giannini D et al. Questionnaires, spirometry and PEF monitoring in epidemiological studies on elderly respiratory patients. Eur Respir J Suppl. 2003 May;40:21s-27s. doi: 10.1183/09031936.03.00402303.
